# Myeloid Krüppel-like factor 2 is a critical regulator of metabolic inflammation

**DOI:** 10.1038/s41467-020-19760-3

**Published:** 2020-11-18

**Authors:** David R. Sweet, Neelakantan T. Vasudevan, Liyan Fan, Chloe E. Booth, Komal S. Keerthy, Xudong Liao, Vinesh Vinayachandran, Yoichi Takami, Derin Tugal, Nikunj Sharma, E. Ricky Chan, Lilei Zhang, Yulan Qing, Stanton L. Gerson, Chen Fu, Anthony Wynshaw-Boris, Panjamaporn Sangwung, Lalitha Nayak, Paul Holvoet, Keiichiro Matoba, Yuan Lu, Guangjin Zhou, Mukesh K. Jain

**Affiliations:** 1 Case Cardiovascular Research Institute, Case Western Reserve University, and Harrington Heart and Vascular Institute, University Hospitals Cleveland Medical Center, Cleveland, OH USA; 2 Department of Pathology, Case Western Reserve University, Cleveland, OH USA; 3 Department of Geriatric Medicine, Osaka University Graduate School of Medicine, Osaka, Japan; 4 Department of Medicine, Cardiovascular Division, Beth Israel Deaconess Medical Center and Harvard Medical School, Boston, MA USA; 5 DBPAP/OVRR/CBER, Food and Drug Administration, Silver Spring, MD USA; 6 Institute for Computational Biology, Case Western Reserve University, Cleveland, OH USA; 7 Department of Molecular and Human Genetics, Baylor College of Medicine, Houston, TX USA; 8 Case Comprehensive Cancer Center, Case Western Reserve University, Cleveland, OH USA; 9 National Center for Regenerative Medicine, Seidman Cancer Center, University Hospitals Cleveland Medical Center and Case Western Reserve University, Cleveland, OH USA; 10 Department of Genetics and Genome Sciences, Case Western Reserve University, and University Hospitals Cleveland Medical Center, Cleveland, OH USA; 11 Department of Physiology and Biophysics, Case Western Reserve University, Cleveland, OH USA; 12 Division of Hematology and Oncology, University Hospitals Cleveland Medical Center, Cleveland, USA; 13 Experimental Cardiology, Department of Cardiovascular Sciences, KU Leuven, Leuven, Belgium; 14 Charles River Laboratories, Ashland, OH USA

**Keywords:** Microglial cells, Type 2 diabetes, Metabolic syndrome, Inflammation

## Abstract

Substantial evidence implicates crosstalk between metabolic tissues and the immune system in the inception and progression of obesity. However, molecular regulators that orchestrate metaflammation both centrally and peripherally remains incompletely understood. Here, we identify myeloid Krüppel-like factor 2 (KLF2) as an essential regulator of obesity and its sequelae. In mice and humans, consumption of a fatty diet downregulates myeloid KLF2 levels. Under basal conditions, myeloid-specific KLF2 knockout mice (K2KO) exhibit increased feeding and weight gain. High-fat diet (HFD) feeding further exacerbates the K2KO metabolic disease phenotype. Mechanistically, loss of myeloid KLF2 increases metaflammation in peripheral and central tissues. A combination of pair-feeding, bone marrow-transplant, and microglial ablation implicate central and peripheral contributions to K2KO-induced metabolic dysfunction observed. Finally, overexpression of myeloid KLF2 protects mice from HFD-induced obesity and insulin resistance. Together, these data establish myeloid KLF2 as a nodal regulator of central and peripheral metabolic inflammation in homeostasis and disease.

## Abstract (web summary)

Inflammation contributes to the development of metabolic disease through incompletely understood mechanisms. Here the authors report that deletion of the transcription factor KLF2 in myeloid cells leads to increased feeding and weight gain in mice with concomitant peripheral and central tissue inflammation, while overexpression protects against diet-induced metabolic disease.

## Introduction

Obesity represents a major health concern that imposes significant risk for related pathologies such as insulin resistance, cardiovascular disease, and fatty liver disease^1^. Accumulating evidence implicates crosstalk between the immune system and metabolic tissues as critical to obesity and its sequelae. Although mechanisms governing immune activation have been shaped by the enormous selection pressure to combat infection over millennia, these pathways can be usurped by noxious metabolites (e.g., saturated fatty acids, oxidized cholesterol, and elevated glucose) that are abundant in states of nutrient excess that characterize contemporary human existence. Indeed, these metabolic metabolites can induce a state of low-grade chronic inflammation (termed as “metaflammation”) by utilizing conserved signaling processes, that link metabolic and inflammatory signaling capable of activating the immune system and modulating metabolic tissue function^2^. Further, heightened inflammation in immune cells both centrally (e.g., brain) and peripherally (e.g., adipose, liver) can contribute to diet-induced metabolic disease. However, few factors have been identified that serve to restrain metaflammation under homeostatic conditions, with even less data demonstrating relative contributions from the central and peripheral myeloid compartments.

Prior work from our group and others has identified the transcription factor Krüppel-like factor 2 (KLF2) as a tonic repressor of myeloid cell activation within the central nervous system (CNS) and periphery^3,4^. Mice with myeloid-specific deletion of KLF2 exhibit spontaneous activation of myeloid cells, increased basal inflammatory cytokine release, and an altered response to infection^4^. In addition, studies in humans have demonstrated that low KLF2 expression is associated with numerous inflammatory conditions, including those with metabolic implications such as coronary artery disease^4,5^. KLF2, therefore, represents an attractive target for studying critical regulators of metaflammation.

In this study, we utilize a murine myeloid KLF2 deletion model to uncover a role for this factor in regulating transcriptional networks and effectors of metabolic inflammation. KLF2 is reduced in response to inflammatory stimuli encountered in obesity, which subsequently permits unrestrained central and peripheral metaflammation, ultimately contributing to obesity and metabolic disease. Furthermore, we demonstrate using numerous models that both central and peripheral myeloid cell populations govern metaflammation and metabolic disease. Finally, we use a myeloid KLF2 overexpressing mouse to demonstrate protection against diet-induced metabolic disease.

## Results

### KLF2 regulates metaflammation transcription in macrophages

**Fig. 1:**
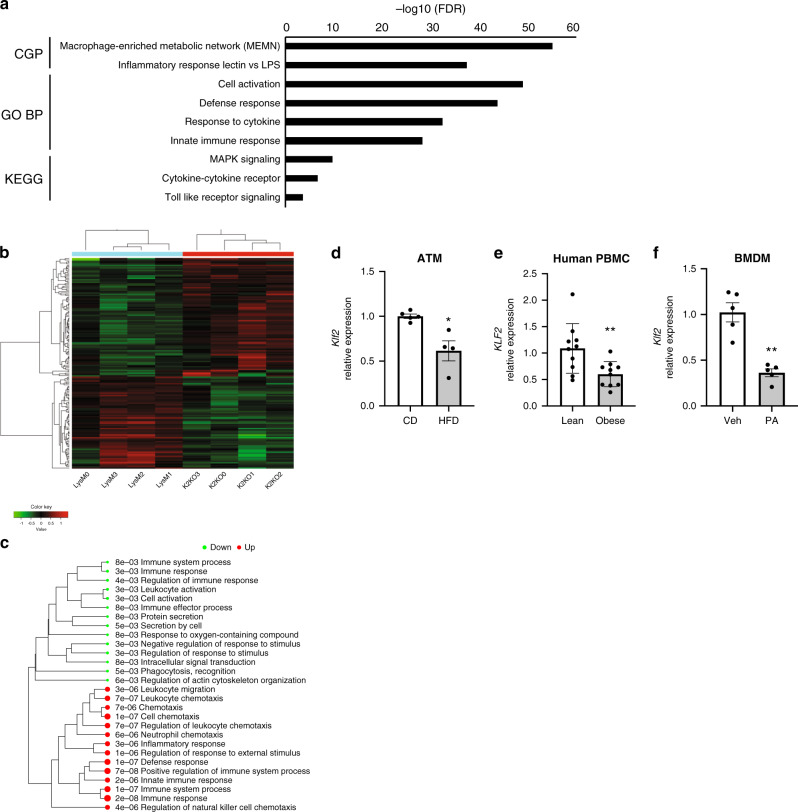
Myeloid KLF2 regulates a macrophage-enriched metabolic network and is responsive to metaflammatory stress. **a** Gene-set enrichment analysis of bone marrow-derived macrophages (BMDMs) from myeloid KLF2 knockout or control mice demonstrating significant enrichment in the macrophage-enriched metabolic syndrome network (MEMN) in addition to several innate immune pathways. Several annotated genesets from the Molecular Signature Database were explored including CGP chemical genetic perturbations, GO BP Gene Ontology biological processes, and KEGG Kyoto Encyclopedia of Genes and Genomes *n* = 4 biological replicates. **b** Heatmap of hierarchal clustering of MEMN genes demonstrating genotype clustering, based on DEGs from *n* = 4 biological replicates. **c** Gene ontology analysis demonstrating up and downregulated genesets for MEMN differentially expressed genes, based on DEGs from *n* = 4 biological replicates. **d** *Klf2* expression from adipose tissue macrophages (ATM) harvested from WT mice fed either control diet (CD, *n* = 5) or high-fat diet (HFD, *n* = 4) for one month, *n* represent biologically independent mice, *p* = 0.0159. **e** *KLF2* expression in human peripheral blood mononuclear cells (PBMC) from lean and obese patients, *n* = 10 biologically independent human subjects, *p* = 0.0068 **f** WT BMDM *Klf2* expression after acute treatment with palmitic acid (PA), *n* = 5 biologically independent mice, *p* = 0.0079. *p < 0.05, **p < 0.01 by unpaired, two-tailed Student’s *t*-test, comparisons marked or indicated in figure legend. Error bars represent SEM. Source data are provided as a Source Data file.

RNA-sequencing of bone marrow-derived macrophages (BMDMs) isolated from myeloid-specific KLF2 knockout (K2KO) and control (LysM) mice provided initial evidence suggesting that myeloid KLF2 serves as a transcriptional regulator of metaflammation. Among the most highly enriched pathways was the macrophage-enriched metabolic network (MEMN), which contains genes shown to have a causal effect on metabolic syndrome (Fig. 1a, see ref. ^6^). Additionally, numerous innate-immune specific transcriptional pathways were enriched (Fig. 1a). Hierarchal cluster heat mapping of MEMN-specific genes demonstrates genotype-dependent clustering in the absence of KLF2 (Fig. 1b). Among the enriched MEMN genes, the most highly expressed in K2KO macrophages were *Vcam1, Cacnb3, Slc8a1, Ccdc102a, Spic, Man2b1, Plac8, Bgn, Layn, Tspan33, and Tgfb3*. Interestingly, the majority of the upregulated genes within the MEMN were those involved in the immune response, cell migration, and cytokine release, further implicating KLF2 in the regulation of metaflammatory processes (Fig. 1c).

Given the widespread transcriptional impact of KLF2 in networks related to obesity and metabolic disease, we sought to determine if diet-induced obesity affects myeloid KLF2 expression in in mammals. We first isolated adipose tissue macrophages (ATMs) from WT mice fed either control diet (CD) or HFD. After one month of HFD, *Klf2* expression in murine ATMs was reduced by 40% (Fig. 1d). This phenomenon was also seen in human patient samples. Peripheral blood mononuclear cells (PBMCs) from lean or obese patients differentially expressed *KLF2*, with obese individuals exhibiting a 40% decrease in expression (Fig. 1e)^7^. Further, *KLF2* expression in humans is inversely associated with numerous inflammatory and metabolic disease metrics, such as *TNFA* expression, soluble C-reactive protein levels, body mass index (BMI), and hypertension (Supplementary Fig. 1A–F). These data suggest that myeloid KLF2 is responsive to excess metabolic stimuli and that the utilization of pathways meant to decrease KLF2 in response to infection may serve as a mechanism of metaflammation induction. To explore this further, we treated macrophages with palmitic acid (PA), a saturated fatty acid that is present at high concentrations in HFD and induces inflammatory activation of macrophages. Indeed, PA-treated WT macrophages decreased expression of *Klf2* more than 50% (Fig. 1f). Previous reports have elucidated mechanisms by which PA activates immune cells that include many signaling pathways shared by pathogen-associated molecular patterns (i.e., lipopolysaccharide, LPS). These include activation of c-Jun N-terminal kinase (JNK) and subsequent NF-κB-mediated transcription^8,9^. Early studies from our group demonstrated that cytokine-mediated downregulation of KLF2 occurs, in part, via NF-κB inhibition of MEF2, a major driving transcription factor of *Klf2* transcription^10^. Like cytokine-mediated repression, PA partially requires NF-κB activity to decrease *Klf2* levels in macrophages (Supplementary Fig. 2A). Additionally, upstream JNK activity participates in PA-mediated *Klf2* suppression, further demonstrating shared signaling pathways that facilitate immunometabolic crosstalk (Supplementary Fig. 2A). Together, these data indicate that conditions known to contribute to obesity are associated with loss of KLF2 expression in macrophages, likely contributing to metaflammatory disease.

### K2KO contributes to basal and diet-induced metabolic disease

**Fig. 2:**
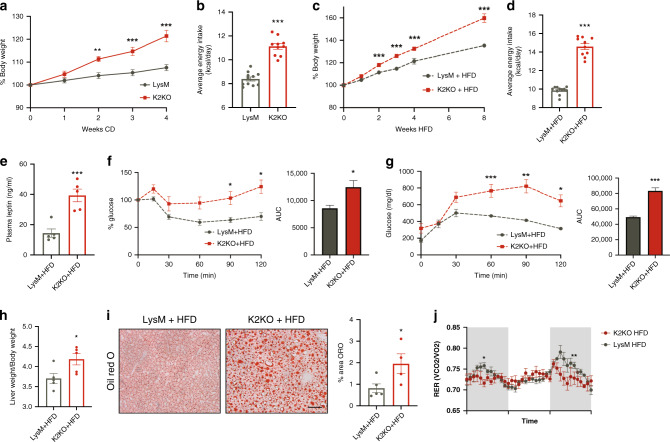
K2KO mice exhibit significant metabolic disease at baseline and with HFD. **a** K2KO mice on control diet (CD) gain weight more rapidly than controls, *n* = 10, 2 weeks *p* = 0.0017, 3 and 4 weeks *p* < 0.0001. **b** K2KO mice have increased daily food intake in basal conditions, LysM *n* = 11, K2KO *n* = 10, *p* < 0.0001. **c** High fat diet (HFD) feeding exaggerates the obesity phenotype seen in K2KO mice, LysM *n* = 10, K2KO *n* = 12, 2 weeks *p* = 0.0083, 3 weeks *p* = 0.0012, 4 weeks *p* = 0.0133, 8 weeks *p* = 0.0038. **d** Increased energy intake in HFD-fed K2KO mice as measured by daily food consumption, *n* = 10, *p* < 0.0001. **e** Increased plasma leptin levels in HFD-fed K2KO mice indicative of increased adiposity and leptin resistance, *n* = 5, *p* = 0.0079. **f** Intraperitoneal insulin tolerance test (IPITT) demonstrates relative insulin resistance in HFD-fed K2KO mice, representative graph of *n* = 4, validated >3 times. AUC, area under curve, *p* = 0.0286. **g** Intraperitoneal glucose tolerance test (IPGTT) reveals relative glucose intolerance in HFD-fed K2KO mice, *n* = 4, validated >3 times, *p* = 0.0009. **h** Liver weight to body weight (BW) ratio demonstrating hepatomegaly in HFD-fed K2KO mice, *n* = 5, *p* = 0.0317. **i** Oil red O (ORO) staining indicating increased lipid accumulation in the livers of K2KO mice fed HFD. Quantification is of proportional area of ORO+ staining, representative images shown, LysM *n* = 5, K2KO *n* = 4, *p* = 0.0456. Scale bar = 100 µm **j** Respiratory exchange ratio (RER) from metabolic cage experiments over multiple light-dark cycles, mice were fed one month HFD, *n* = 4. *p < 0.05, **p < 0.01, ***p < 0.001 by unpaired, two-tailed Student’s *t*-test, comparisons marked or indicated in figure legend. Error bars represent SEM. All mice on were fed CD/HFD for 1 month unless otherwise indicated. Metabolic parameters were also conducted at one month HFD unless otherwise indicated. *n* represents biologically independent mice throughout entire figure. Source data are provided as a Source Data file.

To determine if loss of myeloid KLF2 conferred susceptibility to metabolic disease in vivo, we first characterized the metabolic phenotype of the K2KO mice at baseline. Remarkably, K2KO adult mice weighed more than controls and have significantly more weight gain over time on CD (Supplementary Fig. 2B and Fig. 2a). These weight differences, however, were not seen at weaning (Supplementary Fig. 2C). To explore if differences in weight gain are attributable to altered feeding behavior, we measured average food intake and found that K2KO mice exhibit increased basal energy daily consumption (Fig. 2b). There were comparable levels of circulating leptin (Supplementary Fig. 3A) and a statistically insignificant trend of increased *Lep* mRNA expression in K2KO adipose tissue at baseline (Supplementary Fig. 3B), indicating that increased feeding was not due to low levels of this important satiety-inducing hormone.

While loss of myeloid KLF2 contributes to a significant obesity phenotype at baseline, we sought to explore whether adding a metaflammatory stimulus such as HFD would exacerbate genotype-dependent differences in metabolic disease. Indeed, throughout the HFD regimen, K2KO mice gained weight twice as fast as control mice (Fig. 2c). As with mice on CD, K2KO mice demonstrated higher food intake (Fig. 2d) with higher adipose leptin transcription (Supplementary Fig. 3C). Interestingly, HFD-fed K2KO mice also had more circulating leptin in the plasma (Fig. 2e). Taken together, these data suggest that loss of myeloid KLF2 promotes a leptin-resistant phenotype, likely contributing to obesity in the basal and diet-induced states.

Apart from obesity, HFD and metaflammation can also influence the onset of metabolic diseases such as insulin resistance and nonalcoholic fatty liver disease (NAFLD). To explore whether myeloid KLF2 regulates these pathologies, we first performed intraperitoneal insulin tolerance test (IPITT) and glucose tolerance test (IPGTT), which revealed that HFD-fed K2KO mice are insulin resistant and glucose intolerant, respectively (Fig. 2f, g). This phenomenon occurred in the presence of increased circulating insulin levels (Supplementary Fig. 3D), further demonstrating insulin resistance. These mice also demonstrated NAFLD as indicated by hepatomegaly and increased hepatic lipid deposition (Fig. 2h, i). This metabolic disease phenotype exists independent of significant differences in energy utilization and expenditure or other calorimetric readouts, suggesting that nutrient uptake is likely a major contributor to the K2KO-induced obesity phenotype (Fig. 2j and Supplementary Fig. 4). Together, these results demonstrate that loss of KLF2 in myeloid cells confers a strong propensity towards metabolic disease under basal and an excess nutrient states.

### Enhanced peripheral and central metaflammation in K2KO mice

Given the well-documented role of KLF2 as a nodal regulator of inflammatory activation within myeloid cells^3,4,11,12^, along with evidence that loss of KLF2 activates an inflammatory metabolic syndrome network (Fig. 1a), we sought to discern whether K2KO mice exhibited heightened inflammation at baseline and with HFD stimulus in both central and peripheral compartments.

**Fig. 3:**
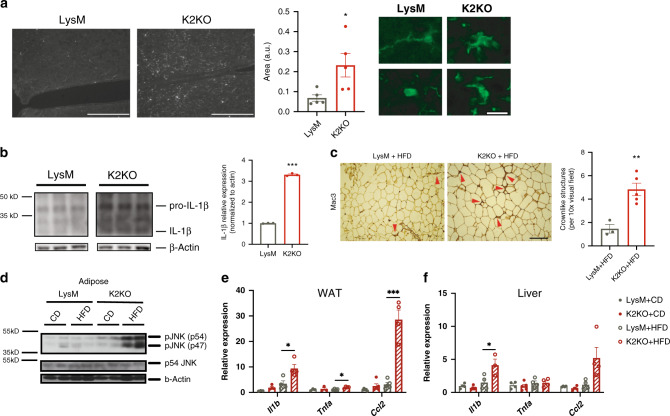
KLF2 regulates central and peripheral metaflammation to resist metabolic disease. **a** K2KO mice on CD have increased microglial activation within the hypothalamus as exhibited by Iba1 proportional area and characteristic morphological changes, representative images shown, *n* = 5, *p* = 0.0277, scale bars: low magnification = 200 µm, high magnification = 20 µm. **b** Western blot on hypothalamic lysates from CD-fed mice demonstrate increased interleukin-1 beta (IL-1β) production in K2KO mice, *n* = 3, *p* < 0.0001. **c** Mac3 staining of adipose tissue from HFD-fed mice reveals K2KO mice have macrophage expansion as indicated by increased crownlike structures, representative image, LysM *n* = 3, K2KO *n* = 5, *p* = 0.0045, scale bar = 100 µm. **d** Western blot of JNK activity demonstrating that month HFD-fed K2KO mice have elevated JNK phosphorylation, representative blot of *n* = 2. **e** Inflammatory gene qPCR from WAT lysate from CD and HFD-fed mice, *n* = 4, *p* values: HFD *Il1b* = 0.029, HFD *Tnfa* = 0.0126, HFD *Ccl2* = 0.0001. **f** Inflammatory gene qPCR from liver lysate from CD and HFD-fed mice, *n* = 4, HFD *Il1b* *p* = 0.0446 by unpaired, two-tailed Student’s *t*-test, comparisons marked or indicated in figure legend. Error bars represent SEM. All mice on were fed CD/HFD for 1 month unless otherwise indicated. *n* represents biologically independent mice throughout entire figure. Source data are provided as a Source Data file.

The hypothalamus is extremely sensitive to metaflammatory stimuli, serving as an early responder to dietary stress. Inflammation within the hypothalamus is an important cause of leptin resistance and increased feeding behavior^13^. Because of this, hypothalamic inflammation is associated with metabolic disease in rodents and humans^14^. Remarkably, microglia in K2KO mice assumed an activated morphology within the hypothalamus under basal conditions (Fig. 3a). Increased interleukin-1β (IL-1β) within the hypothalami of K2KO mice provided additional evidence of neuroinflammation at baseline (Fig. 3b). Given the robust microglial response seen in K2KO mice, we believe that KLF2 serves to regulate aberrant activation of hypothalamic microglia, thereby protecting against centrally derived metabolic disease.

In addition to central inflammation, inflammation in peripheral metabolic organs such as white adipose tissue (WAT) and liver can contribute to obesity and metabolic disease^15,16^. To determine if loss of KLF2 leads to metabolic tissue macrophage expansion, we first stained WAT for macrophage marker Mac3. After HFD, K2KO mice exhibited increased presence of crown-like structures (CLS) that are indicative of macrophages juxtaposed against adipocytes (Fig. 3c). This was in conjunction with increased transcriptional expression of macrophage markers (e.g., *Cd68, F4/80*, and *Cd64*) within WAT and liver (Supplementary Fig. 5A, B).

An important component of the metaflammation model is conserved pathway utilization between the immune and metabolic systems^2^. JNK is an important inflammatory stress kinase and has been shown to contribute to obesity and insulin resistance^17^. We, therefore, sought to explore whether loss of myeloid KLF2 would increase activity of this metaflammatory kinase in WAT. Indeed, HFD-fed K2KO mice had substantially higher JNK activation, providing additional evidence of metabolic tissue inflammation (Fig. 3d). Further, transcriptional levels of inflammatory cytokines were increased in WAT and liver of K2KO mice after HFD (Fig. 3e, f). Collectively, these data demonstrate that loss of myeloid KLF2 leads to central and peripheral metaflammation.

### Central metaflammation contributes to disease in K2KO mice

**Fig. 4:**
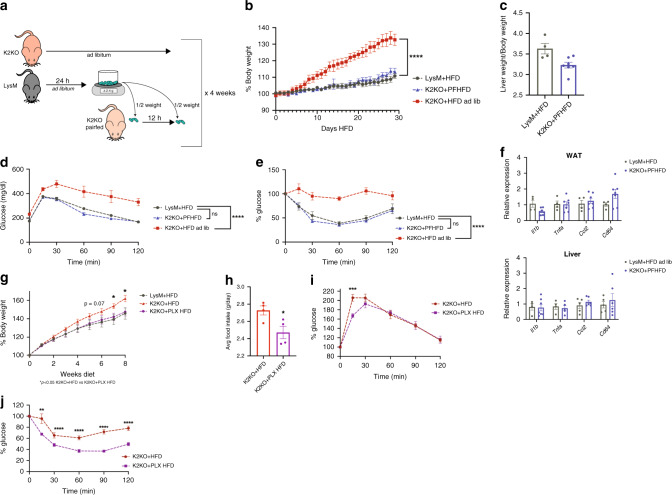
Loss of myeloid KLF2 affects centrally-governed food intake and metabolic disease. **a** Schematic describing pair-feeding protocol. **b** Pair-feeding K2KO mice (K2KO+PFHFD) abrogates weight gain compared to K2KO mice fed HFD ad libitum, LysM and K2KO ad lib. *n* = 4, K2KO PFHFD *n* = 7, ****p < 0.0001 comparing K2KO PFHFD to K2KO HFD by repeated measures one-way ANOVA with Tukey’s multiple comparison test. **c** Pair-feeding attenuates K2KO hepatomegaly, LysM *n* = 4, K2KO PFHFD *n* = 7. **d** Intraperitoneal glucose tolerance test (IPGTT) demonstrates improved glucose sensitivity in one month K2KO PFHFD mice, LysM and K2KO ad lib. *n* = 4, K2KO PFHFD *n* = 7, ****p < 0.0001 comparing K2KO PFHFD to K2KO HFD by repeated measures one-way ANOVA with Tukey’s multiple comparison test. **e** Intraperitoneal insulin tolerance test (IPITT) demonstrates improved insulin sensitivity in one month K2KO PFHFD mice, LysM and K2KO ad lib. *n* = 4, K2KO PFHFD *n* = 7, ****p < 0.0001 comparing K2KO PFHFD to K2KO HFD by repeated measures one-way ANOVA with Tukey’s multiple comparison test. **f** Pair-feeding K2KO mice rescues inflammatory gene transcription in WAT and liver, LysM and K2KO ad lib. *n* = 4, K2KO PFHFD *n* = 7. **g** Microglial ablated K2KO mice on HFD (using PLX5622 formulated HFD, PLX HFD) demonstrate improved weight gain over 8 weeks, LysM *n* = 7, K2KO+HFD and K2KO+PLXHFD *n* = 12, 7 weeks *p* = 0.0153, 8 weeks *p* = 0.0015 comparing K2KO+HFD to K2KO+PLX HFD. **h** Two months K2KO PLX HFD-fed mice consume less food than K2KO HFD mice, *n* = 4, *p* = 0.0284. **i** IPGTT demonstrates rescued glucose tolerance in K2KO PLX HFD mice, *n* = 12, *p* = 0.0042 at 15 min. **j** IPITT demonstrates rescued insulin sensitivity in K2KO PLX HFD mice, *n* = 12, *p* = 0.0482 (15 min), 0.0371 (30 min), 0.0003 (60 min), <0.0001 (90 min), 0.0001 (120 min) by unpaired two-tailed Student’s *t*-test, comparisons marked or indicated in figure legend. Error bars represent SEM. Pair-feeding experiments and associated metabolic parameters were performed for/at 1 month. PLX HFD metabolic parameters were performed at 1 month unless otherwise indicated. *n* represents biologically independent mice throughout entire figure. Source data are provided as a Source Data file.

While the contributions of central and peripheral myeloid cells to metabolic disease have been studied separately, little work has been done to decipher the relative importance of these populations together within metaflammatory models. To determine the extent to which central myeloid cells and feeding behavior contribute to the development of metabolic disease in the context of KLF2 deletion, we employed two methods of central nervous system (CNS) macrophage manipulation. To control for the effects of increased food intake on the K2KO phenotype (Fig. 2b), we utilized a pair-feeding model in which the amount of HFD given to K2KO mice was limited to the amount of food consumed by LysM mice for the previous day. In order to control for prolonged periods of fasting, we divided these feeding events into two meals (Fig. 4a). Consistent with our hypothesis that increased food intake is a major driver of K2KO-mediated obesity, we found that pair-feeding K2KO mice completely abrogated the weight gain differences on HFD (Fig. 4b). Additionally, pair-feeding rescued K2KO mice from accelerated metabolic disease including hepatomegaly, glucose intolerance, and insulin resistance (Fig. 4c–e). This demonstrated that controlling for food intake likely mitigates the effects of microglial-mediated metaflammation. We investigated if the protection against metabolic disease with pair-feeding was also associated with decreased peripheral metaflammation. Pair-fed K2KO mice exhibited similar levels of inflammatory transcription in WAT and liver as LysM mice fed HFD ad libitum (Fig. 4f), demonstrating that, despite the presence of peripheral K2KO myeloid cells, controlling food intake is sufficient to protect against peripheral metaflammatory change.

Although pair-feeding underscores the importance of processes downstream of microglial activation on metabolic disease, we wanted to determine if activation of microglia by loss of KLF2 was a major driver of food intake, obesity, and diabetes. To study this, we utilized the experimental CSF1R inhibitor PLX5622, which ablates microglia and can be sustained over long experimental timelines^18^. After first ablating microglia for 21 days using a CD formulated with PLX5622, we then fed K2KO mice HFD formulated to include PLX5622 (PLX HFD) for 8 weeks to observe weight changes. Histological analysis confirmed substantial depletion of Iba1+ microglia from K2KO hypothalami (Supplementary Fig. 6). Interestingly, while a WT PLX HFD-fed control demonstrated complete ablation of microglia, K2KO mice still had remaining cells that were morphologically distinct from those normally found in K2KO hypothalami, suggesting a unique population that is resistant to CSF1R inhibition (Supplementary Fig. 6). Consistent with pair-feeding, ablation of K2KO microglia reduced the weight gain to that of LysM HFD mice (Fig. 4g). These mice also exhibited less food intake compared to K2KO HFD mice, providing a potential mechanism of protection against weight gain (Fig. 4h). Furthermore, K2KO PLX HFD mice were more glucose tolerant and insulin sensitive compared to K2KO HFD mice, confirming a deleterious role for K2KO microglia on metabolic homeostasis (Fig. 4i, j).

### Loss of myeloid KLF2 in periphery affects metabolic disease

**Fig. 5:**
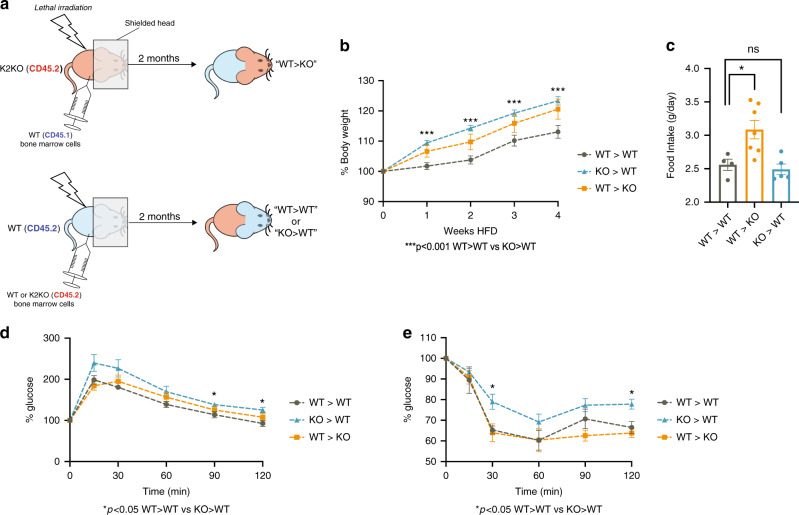
Deletion of KLF2 confined to peripheral myeloid cells contributes to metabolic disease. **a** Schematic depiction of bone marrow transplant protocol and resultant chimeras created. **b** Chimeras with preserved WT microglia but K2KO hematopoietic myeloid cells (KO>WT) demonstrate elevated body weight gain on 4 weeks HFD, WT>WT *n* = 9, WT>KO *n* = 10, KO>WT *n* = 13, *p* = 0.0002 (1 week), <0.0001 (2 weeks), 0.0020 (3 weeks), 0.0028 (4 weeks) comparing WT>WT to KO>WT. **c** Chimeras with preserved KO microglia but WT hematopoietic myeloid cells (WT>KO) demonstrate elevated food intake across one month HFD, WT>WT *n* = 4, WT>KO *n* = 7, KO>WT *n* = 5, *p* = 0.0251. (**d**) KO > WT mice have slightly worse glucose intolerance on GTT, *n* = 5 (*p* = 0.0202 at 90 min, 0.0109 at 120 min) and (**e**) worse insulin sensitivity on ITT, *n* = 5, *p* = 0.0111 (15 min) and 0.0177 (120 min) by unpaired, two-tailed Student’s *t*-test, comparisons marked or indicated in figure. Error bars represent SEM. *n* represents biologically independent mice throughout entire figure. Source data are provided as a Source Data file.

Although studies in Fig. 4 demonstrate a significant role of CNS myeloid cells in the genesis of metabolic disease in K2KO mice, we could not rule out a contribution of peripheral cells in facilitating metaflammation. To examine whether loss of KLF2 in peripheral, hematopoietically-derived myeloid cells contributes to metabolic disease, we generated bone marrow chimera mice in which central myeloid cells were spared from radiation using head shielding. After irradiation, WT or K2KO mice were constituted with either WT or K2KO bone marrow (Fig. 5a). After 2 months of reconstitution (Supplementary Fig. 7), we subjected mice with K2KO central cells but WT peripheral cells (WT>KO), mice with central WT cells but K2KO peripheral cells (KO>WT), and WT>WT controls to 4 weeks of HFD. Although WT>KO mice demonstrate a slightly elevated weight gain, KO>WT mice generated a more robust weight gain phenotype (Fig. 5b). This occurred despite no differences in food intake compared to WT>WT mice (Fig. 5c). Although PLX5622 studies would suggest that K2KO microglia are major drivers of metabolic disease through increased food intake, WT>KO mice weighed only modestly more than controls despite increased food intake. Peripheral K2KO myeloid cells also contribute to dysregulated glucose homeostasis as evidenced by IPGTT and IPITT (Fig. 5d, e), further underscoring a substantial role for peripheral loss of myeloid KLF2 in metabolic disease.

### Overexpression of myeloid KLF2 protects against disease

**Fig. 6:**
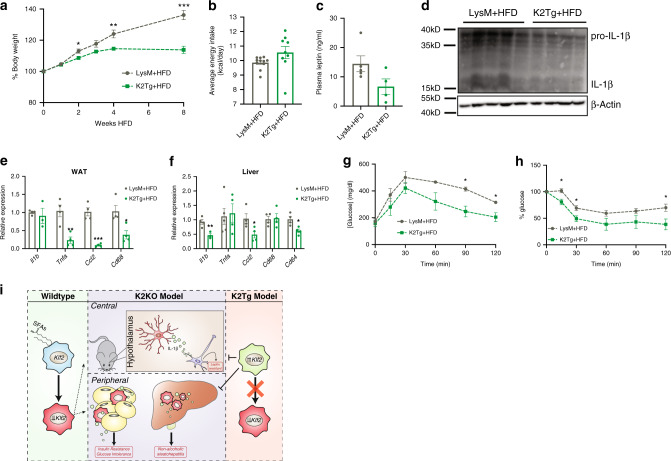
Overexpression of myeloid KLF2 protects against metabolic disease. **a** Myeloid KLF2 overexpressing mice (K2Tg) are protected against HFD-induced weight gain, *n* = 15, *p* = 0.0253 (2 weeks), 0.00238 (4 weeks), 0.005 (8 weeks). **b** K2Tg mice consume similar amounts of food daily compared to LysM mice across a HFD regimen, LysM *n* = 11, K2Tg *n* = 9. **c** K2Tg have diminished plasma leptin levels after HFD, LysM *n* = 5, K2Tg *n* = 4. **d** Western blot on hypothalamic lysates from HFD-fed mice demonstrate decreased interleukin-1 beta (IL-1β) production in K2Tg mice, LysM *n* = 5, K2Tg *n* = 4. **e** K2Tg have decreased inflammatory gene expression in WAT after HFD, *n* = 4, *p* values: *Tnfa* = 0.0046*, Ccl2* = 0.00019, *Cd64* = 0.0168. **f** K2Tg have decreased inflammatory gene expression in liver after HFD, *n* = 4, *p* values: *Il1b* = 0.0099, *Ccl2* = 0.0394, *Cd64* = 0.0155. **g** IPGTT and **h** IPITT demonstrating improved glucose handling by K2Tg after HFD, LysM *n* = 4, K2Tg *n* = 5. **i** Working model describing how myeloid KLF2 expression affects metabolic disease centrally and peripherally. *p < 0.05, **p < 0.01, ***p < 0.001 by unpaired two-tailed Student’s *t*-test. Error bars represent SEM. All mice on were fed HFD for 1 month unless otherwise indicated. *n* represents biologically independent mice throughout entire figure. Source data are provided as a Source Data file.

Our data suggest that downregulation of myeloid KLF2 may serve as a metaflammatory switch that triggers metabolic disease in response to HFD. Given this, we hypothesized that if myeloid KLF2 expression were maintained despite HFD stimulus, mice would be protected against obesity and metabolic disease. To explore this, we generated a mouse model with sustained overexpression of myeloid KLF2 (K2Tg) (Supplementary Fig. 8A, B). After feeding K2Tg and control mice HFD, we noted that K2Tg mice weight gain rate plateaued over time, while control mice continued to gain weight rapidly (Fig. 6a). Interestingly, K2Tg mice consumed HFD at an equivalent rate as LysM mice, but had lower circulating leptin levels, indicating a possible leptin sensitivity of these mice (Fig. 6b, c). Given the elevated metaflammation seen in the hypothalamus of K2KO (Fig. 3b), we sought to determine if K2Tg mice were protected against diet-induced IL-1β induction. Indeed, less IL-1β was detected in HFD-fed K2Tg hypothalmi, demonstrating protection from diet-induced central metaflammation. In addition to this protection, K2Tg exhibited decreased evidence of peripheral metaflammation as well (Fig. 6e, f). Further, K2Tg were protected against diet-induced insulin resistance and glucose tolerance as demonstrated on IPITT and IPGTT (Fig. 6g, h).

Collectively, these studies describe a model of metabolic inflammation and disease that centers around KLF2 expression within myeloid cells. In response to fatty acids seen in HFD (e.g., PA), myeloid cells decrease KLF2 expression, resulting in unrestrained inflammatory activation. Centrally, this contributes to microglial-induced leptin resistance and increased food intake. Concurrently, peripheral loss of myeloid KLF2 contributes to metabolic disease. Finally, sustained overexpression of KLF2 in myeloid cells protects against both central and peripheral metaflammation and metabolic disease (Fig. 6i).

## Discussion

KLFs are well-documented to be key effectors of both metabolism and inflammation^19,20^. That this diverse family of transcription factors appear to have evolved, in part, to be central in the immunometabolic crosstalk seen in metabolic disease further underscores the interconnectedness of these two fundamental processes. While recent work has identified myeloid specific regulators of metabolic disease^21,22^, few have demonstrated a role of regulating basal metaflammation. There is a large body of work demonstrating that KLF2 serves as a dominant repressor of macrophage activation in acute and chronic inflammatory states, whose expression maintains quiescence of these cells during basal states^4,5,11,12,23^. This study further establishes the importance of myeloid KLF2 in maintaining quiescence of inflammatory networks in basal and obesogenic contexts. Loss of myeloid KLF2 caused mice to exhibit early stages of metabolic disease without HFD stimulation, demonstrating the importance of this factor in maintaining homeostatic control over metaflammation. This study also further demonstrates the profound sensitivity the hypothalamus has to metaflammation. The continual low-grade inflammation seen with microglial deletion of KLF2 was sufficient to affect feeding behavior, even before peripheral metabolic tissues demonstrated overt evidence of metaflammatory disease. This corroborates previous reports that showed hypothalamic inflammatory gene transcription after only one week of HFD^14^. Consistently, ablation of K2KO microglia significantly impacted weight gain and glucose homeostasis, underscoring that microglial activation via loss of KLF2 drives CNS-mediated metabolic disease. Additional investigation examining inflammatory regulation within the hypothalamus will be crucial in better understanding physiological regulation of energy intake.

Centrally located myeloid cells are not the only KLF2-regulated population that affect this pathology, however. While pair-feeding studies and microglial ablation suggest that heightened food intake stemming from hypothalamic inflammation is a major driver of K2KO-mediated disease, bone marrow chimera studies indicate that hematopoietic myeloid cells can contribute to obesity and diabetes as well, independent of food intake. A number of considerations may account for disparity in results. A caveat to pair-feeding studies is that there is a near unavoidable period of fasting between, when pair-fed mice finish their allotted evening meal and that which is given in the morning. Recent evidence suggests that even short-term fasting of 4 h is capable of reducing levels of pro-inflammatory Ly6C^hi^ monocytes in circulation, WAT, and liver^24^. While the major effect of pair-feeding is to normalize food intake, there are potential off-target effects on myeloid cell populations that may complicate conclusions drawn from these studies. There is a large body of literature demonstrating the role of resident and infiltrating macrophages of WAT and liver in interorgan crosstalk in metabolic disease (reviewed in the ref. ^25^). Activated resident and infiltrating ATMs participate in lipolysis that leads to ectopic lipid deposition, contributing to NAFLD, and diabetes^26,27^. Indeed, accumulation of macrophages around apoptotic adipocytes (forming CLS), leads to an increase in macrophage and neutrophil recruitment to the liver, contributing to metabolic disease^26,28^. Given the expansion of WAT macrophages in K2KO mice, along with the tonically active state of these cells, we posit that peripheral K2KO cells contribute to metabolic disease by participating in trafficking of lipotoxic mediators. This model would be in agreeance with bone marrow chimera data in which the presence of activated K2KO myeloid cells in the periphery is sufficient to drive metabolic disease. Additional studies will be required to uncover the specific mechanisms by which loss of KLF2 in myeloid cells affects nutrient handling in peripheral metabolic tissues.

Decreased KLF2 expression has been noted in immune cells in a multitude of inflammatory diseases in humans such as sepsis and coronary artery disease^4,5^. In our model, obesity was associated with diminished KLF2 transcription in humans and mice and, therefore, may reveal a mechanism by which metaflammation arises (Fig. 6i). We hypothesize that HFD metabolites such as saturated fatty acids lead to decreased KLF2 transcription, thereby releasing KLF2-mediated transcriptional regulation of inflammatory genes. Prohibiting the initial diet-induced reduction in KLF2 expression appears to protect against obesity and insulin resistance as demonstrated by the K2Tg mouse model. Loss of KLF2 in macrophages is associated with over 2600 differentially expressed genes, many of which are involved in inflammatory activation of innate immune cells. Given this widespread release of inflammatory gene regulation, we hypothesize that K2KO-mediated activation and disease is likely due to the perturbation of multiple inflammatory signaling pathways, rather than individual mechanisms of activation. The downstream effects of this transcriptional upheaval are exemplified by previous work demonstrating increased circulating levels of nearly every major inflammatory cytokine in K2KO mice at baseline^4^. There are widespread networks that integrate metabolic and inflammatory signaling that ultimately converge on a subset of transcription factors to govern metaflammatory activation. Uncovering these central effectors of metaflammation will have a profound impact on understanding immunometabolic crosstalk while providing potential targets of therapy.

## Methods

### Mice

Myeloid specific KLF2 knockout (K2KO) mice were generated by mating lysozyme M driven Cre recombinase (LysM) mice with floxed *Klf2*^*fl/fl*^ mice to generate deletion of *Klf2* restricted to the myeloid compartment in progeny. Myeloid specific KLF2 transgenic (K2Tg) mice were generated mating LysM mice with mice with a ubiquitin C promoter, followed by a floxed stop codon in front of the KLF2 gene. Upon recombination, the stop codon is removed, allowed constitutive expression of KLF2 in myeloid cells. For wean weight analysis, mice were weighed at 3 weeks of age when weaned from parent cage. For high-fat diet studies, age-matched male mice were randomly allocated to a diet regimen. For all high-fat diet studies unless otherwise indicated (i.e., bone marrow chimera studies), mice were started on diet at 8 weeks of age. All mouse colonies were maintained in a clean animal facility. All mice used were on a C57BL/6J background. Facility housing mice was temperature and humidity controlled, specific pathogen free. Mice had ad libitum access to water and laboratory rodent chow unless otherwise noted in the manuscript (CD/HFD studies, pairfeeding studies), and were exposed to a 12 h light/dark cycle. All animal studies were performed in accordance with national and institutional ethical guidelines and were approved by the Case Western Reserve University IACUC.

### High-fat diet studies

Mice were fed either high-fat diet containing 60% kcal fat (HFD, D12492; Research Diets, New Brunswick, NJ) or control diet containing 10% kcal fat (CD, D12450J; Research Diets). An 8-week diet regimen was used for weight gain studies, with all other diet studies lasting 4 weeks (1 month) unless otherwise noted in figures. Weight gain is presented as a percentage of the baseline weight for each mouse to control for variability between animals. Food intake was measured as the mass of food consumed per mouse, multiplied by the kcal/g value for each diet (3.85 kcal/g for CD, 5.24 kcal/g for HFD) and divided by number of days. For PLX5622 HFD and bone marrow chimera studies, food intake is presented as mass of food consumed per mouse per day.

### Histology and immunostaining

Tissues were harvested and fixed using 10% neutral buffered formalin for 24 h. For cryosections, tissues were transferred to 15% sucrose/PBS solution for 24 h at 4 °C and subsequently transferred to 30% sucrose/PBS solution for 24 h at 4 °C. Tissues were then embedded in Optimal Cutting Temperature (OCT) compound (Fisher Scientifict, Hampton, NH) at −20 °C and sectioned using a cryostat (Leica, Wetzlar, Germany). For immunostaining, cryosections were washed with PBS for 10 min and subsequently blocked with 5% normal goat serum in 0.1% Triton X-100 in PBS for 1 h at room temperature. Primary antibodies were then applied overnight at 4 °C. Sections were then washed three times with PBS and appropriate secondary antibodies were added for 1 h at room temperature. Following three PBS washes, sections were covered in mounting media containing Dapi (Vector, Burlingame, CA) and coverslipped. For paraffin-embedded tissues, standard procedures were used to process and embed. For WAT macrophage staining, paraffin embedded tissues were section at 6 μm, stained with rat anti-CD107b (Mac-3) (1:500, BD Biosciences, San Jose, CA, 550292) For hypothalamus Iba-1 stain, frozen brains were sectioned at 10 μm and stained using rabbit anti-Iba-1 (1:1000, Wako, Richmond, VA, 019-19741) and then Alexa 488 goat anti-rabbit (1:1000). Quantification was performed by measuring Iba-1+ area using ImageJ (NIH). For liver analyses, frozen liver sections (8 μm) were stained using Oil Red O (ORO) according to standard procedures. Area of ORO+ signal was quantified using ImageJ (NIH). For all histological analyses, the analyzer was blinded to the identity of each section.

### Measuring plasma leptin and insulin levels

Plasma from mice on one month HFD/CD was isolated and leptin levels were assayed by ELISA (EMD Millipore, Billerica, MA). Similarly, insulin levels were assessed using ELISA (Crystal Chem, Grove, IL). ELISA analyses were performed according to manufacturers’ instructions.

### Hypothalamic IL-1β analysis

For hypothalamic IL-1β western blots, the hypothalamus was dissected from surrounding brain parenchyma and homogenized in RIPA buffer containing protease and phosphatase inhibitors (Fisher). Equal amounts of protein were resolved using SDS-Page and subjected to immunoblotting using the following antibodies: goat anti-IL-1β (1:1000, R&D Systems, Minneapolis, MN AF-401), and mouse anti-Actin (1:5000, Santa Cruz, Dallas, TX, sc-47778). Blots were quantified using densitometry (ImageJ). IL-1 β was normalized to Actin bands.

### Intraperitoneal glucose tolerance test (IPGTT) and insulin tolerance test (IPITT)

For IPGTT, mice were fasted for 5 h and blood glucose levels were measured. Following an injection of glucose i.p. (1.5 g/kg bodyweight), glucose was measured at 15 min intervals up to 120 min. For IPITT, mice were fasted for 5 h and glucose was measured at 15 min intervals following an injection of insulin i.p. (0.75 U/kg bodyweight). Area under the curve was calculated using the trapezoid method in Excel (Microsoft).

### RNA-Seq and bioinformatics analysis

Bone marrow-derived macrophages (BMDMs) were isolated from LysM and K2KO mice at 3 months of age by flushing femurs and tibia with cold PBS and culturing single cell suspensions in L929 fibroblast-conditioned DMEM media with 10% FBS. Four mice per group were used in sequencing experiment. RNA isolation was performed using the RNeasy kit (Qiagen, Hilden, Germany). cDNA sequencing libraries were prepared using the Illumina TruSeq Stranded Total RNA kit and sequenced on a Illumina HiSeq 2500 platform. Reads from each sample were aligned to the mouse genome (UCSC mm10) using TopHat2^29^. Macrophage gene expression was quantitated as fragments per kilobase of transcript per million mapped reads (FPKM) using Cufflinks^30^. A total of 1465 differentially expressed genes (DEGs) were identified and were defined as genes having absolute log2 fold change > 0.26 (1.2 fold change), and an adjusted *p*-value cutoff *q* < 0.05. Gene set enrichment analysis was performed using GenePattern and the Gene Set Enrichment Analysis (GSEA) toolset and the Molecular Signature Database (MSigDB) from the Broad Institute, which identified significantly enriched pathways and gene ontology terms using a FDR < 0.05 significance cutoff. For hierarchal heatmap clustering, DEGs from the MEMN geneset were input into iDEP^31^. Additional gene ontology analyses based on this heatmap were also performed using iDEP.

### Real-time quantitative RT-PCR

Total RNA was extracted from WAT and Liver via homogenization in TRIzol (Invitrogen, Carlsbad, CA). Alternatively, RNA was isolated from cell culture lysates using commercially available kits (Roche). Two microgram of total RNA was converted to cDNA using iScript cDNA synthesis kit (BioRad, Hercules, CA). Real-time PCR was then performed using Fast SYBR Green Master Mix (Life Technologies, Carlsbad, CA) or, alternatively, Taqman with appropriate probes (Thermo Fisher) on a Step One Real-Time PCR System (Applied Biosystems, Foster City, CA) using gene-specific primers. Technical replicates (multiple wells from the same biological sample) were used to ensure minimal variation within biological samples. Relative expression differences between biological groups were calculated using the ΔΔCT method. Primers used include: *Il-1b* (forward: AGTTGACGGACCCCAAAAG, reverse: AGCTGGATGCTCTCATCAGG), *Tnf-a* (forward: TGCCTATGTCTCAGCCTCTTC, reverse: GAGGCCATTTGGGAACTTCT), *Mcp-1* (forward: GATCATCTTGCTGGTGAATGAGT, reverse: CATCCACGTGTTGGCTCA), *Cd68* (forward: CTTCTGCTGTGGAAATGCAA, reverse: AGAGGGGCTGGTAGGTTGAT), *Cd64* (forward: AGGTTCCTCAATGCCAAGTGA, reverse: GCGACCTCCGAATCTGAAGA), *Klf2* (forward: TTCGGTCTTTTCGAGGACGC, reverse: TCTTGCCGCAGTTGGTGTAG) and *F4/80* (forward: CTTTGGCTATGGGCTTCCAGTC, reverse: GCAAGGAGGACAGAGTTTATCGTG). *Gapdh* (forward: TGTCCGTCGTGGATCTGAC, reverse: CCTGCTTCACCACCTTCTTG) was used as a control gene. Human primers used for PBMC analysis include: *hKLF2* (forward: TGGGCATTTTTGGGCTACCT, reverse: GCTGCCCTCCATCAAACTCT), *hGAPDH* (forward: AATCCCATCACCATCTTCCA, reverse:

TGGACTCCACGACGTACTCA).

### In vivo JNK activity analysis

For adipose JNK activity, WAT from LysM/K2KO CD/HFD-fed mice was lysed and equal amounts of protein were immunoblotted after SDS-PAGE resolution. Antibodies used for immunoblotting are as follows: rabbit anti-p-JNK (1:2000, EMD Millipore, 07-175), rabbit anti-SAPK/JNK (1:2000, Cell Signaling, 9252 S), mouse anti-actin (1:5000, Santa Cruz, sc-47778).

### Palmitic acid-treated macrophages

Palmitic acid (PA) was dissolved in DMSO to a concentration of 20 mM. PA was then conjugated in DMEM with 1% fatty acid-free BSA to a final concentration of 300 μM. BMDMs or RAW264.7 cells were treated for 5 h and then washed with warm PBS before harvesting RNA. Fatty acid-free BSA DMEM was used as a vehicle control. For NF-κB inhibitor studies, RAW 264.7 cells were pre-treated with SN50 (50 μg/mL) for 1 h prior to the addition of either BSA or PA as described above. For JNK inhibitor studies, RAW 264.7 cells were co-treated with BSA, PA, or PA+SP600125 (20 μM) for 5 h as described above.

### Adipose tissue macrophage (ATMs) isolation

ATMs were isolated by first collecting visceral adipose tissue euthanized mice into ice-cold PBS. The tissue was mechanically dissociated by mincing using and subsequently enzymatically dissociated using collagenase dissection. The slurry was filtered and centrifuged to separate adipocytes from the stromal vascular cells (SVCs). After washing the SVCs, cells were incubated with CD11b-positive selection beads (Miltenyi) and separated on a magnetic column. CD11b+ cells were then lysed in RNA lysis buffer (Roche) for downstream qPCR.

### PLX5622 diet experiments

PLX5622 (Plexxikon) is a selective CSF1R inhibitor that leads to the ablation of microglia. PLX5622 was formulated into D12450J 10% kcal from fat control diet (PLX CD, Research Diets, Inc) and into D12492 60% kcal from fat high-fat diet (PLX HFD, Research Diets, Inc). All mice to receive PLX HFD were first given PLX CD for three weeks to reach ablation prior to the addition of HFD. Mice not receiving PLX HFD were first acclimated to CD for three weeks. After 3 weeks of CD with confirmed microglial ablation, PLX HFD experiments were carried out for 8 weeks. Food intake was measured for each cage and divided by the number of mice per cage.

### Bone marrow chimera

WT/K2KO bone marrow chimeras were created utilizing the CD45.1/CD45.2 differences between strains. Recipient mice were placed with the head blocked by a lead block such that the brain would be relatively spared from lethal gamma irradiation. After irradiation, 2 million bone marrow cells from donor mice were transplanted via retro-orbital injections and recipients were allowed to recover for 2 months before blood test of CD45.1/CD45.2 status of circulating immune cells. Blood (50 μL) was drawn from the tail and stained with fluorescent antibodies for CD45.1 and CD45.2 for FACS analysis. Donor/recipient mice combinations are as follows: WT (CD45.1) mice receiving K2KO (CD45.2) bone marrow (KO>WT), K2KO (CD45.2) mice receiving WT (CD45.1) bone marrow (WT>KO), and WT (CD45.2) mice receiving WT (CD45.1) bone marrow (WT>WT).

### Pair-feeding experiments

Pair-feeding experiments were performed according to guidelines and caveats laid out in Ellacott et al.^32^. Mice were individually housed to accurately measure food intake per mouse and to reduce the effects of social hierarchy. To reduce stress, each mouse was housed with a plastic hut. Food intake by LysM+HFD was measured daily and averaged across the entire experimental group (i.e., across all LysM HFD mice). The following day, K2KO pair-fed mice (K2KO PFHFD) were given the average amount of food consumed by LysM HFD mice on the previous day, broken up into two installments to prevent long-term fasting (one at the beginning of the light cycle, one at the beginning of the dark cycle). As a control, a K2KO HFD ad libitum group was included in which singly-house K2KO mice were able to eat as much HFD as desired. Body weight measurements were performed just before the beginning of the dark cycle feeding to limit the effects of the slightly longer fasting time seen between the dark cycle and light cycle feedings. On days in which GTT and ITT were performed, pair-fed mice were given half of their normal light cycle meal one hour before the scheduled five hour fast to equilibrate the fasting of pair-fed and non-pair-fed mice. Pair-fed mice consistently finished this half-meal prior to the five hour fast.

### Indirect calorimetry

Measurements were performed using the Promethion system (Sable Systems International) at Case Western Reserve University MMPC. Mice were individually housed in metabolic chambers with food and tap water ad libitum. Animals were acclimated for 24–36 h. VO_2_ and VCO_2_ of individual mice were measured using metabolic chambers over several 12 h light-dark cycles, and RER was calculated by VCO2/VO2.

### Figure construction

Images were cropped and figures were assembled using Adobe Photoshop and Illustrator (CC 2019), respectively.

### Statistics

Data are shown as mean ± SEM. *p* values for most studies were calculated by unpaired two-tailed Student’s *t*-test (indicated in figure legends). For pair-feeding studies, significance was determined using a repeated measures one-way ANOVA with Tukey’s multiple comparison test. For regression analyses in human samples (Supplementary Fig. 1), simple linear regression was applied to the dataset. Goodness of fit is shown as *R*-squared and *p*-values represent whether the slope of the line of best-fit is significantly non-zero (as determined by Graphpad Prism). *p* values less than 0.05 were considered to be statistically significant.

### Study approval

All animal studies were approved by the Case Western Reserve University’s Institutional Animal Care and Use Committee. Human PBMC cDNA was a generous gift from Dr. Paul Holvoet and colleagues from a previous study (Hulsmans et al.^7^). The original study, which ascertained the samples, complies with the Declaration of Helsinki, and the locally appointed Ethics Committee (KU Leuven) approved the study protocol. All human participants gave written informed consent for qPCR analyses of cell samples. Further, assaying for *KLF2* expression has been verified and approved by the Ethics Committee at UZ/KU Leuven.

### Reporting summary

Further information on research design is available in the Nature Research Reporting Summary linked to this article.

## Supplementary information

Supplementary Information

Reporting Summary

## Data Availability

The RNA sequencing data discussed in this publication have been deposited in NCBI’s Gene Expression Omnibus and are accessible through GEO Series accession number GSE149119. Source data for all applicable figures are provided in this paper. Databases and publicly available analysis software used in this manuscript include the Molecular Signature Database (MSigDB, https://www.gsea-msigdb.org/gsea/index.jsp), the Kyoto encyclopedia of genes and genomes (KEGG, https://www.genome.jp/kegg), iDEP.91 (http://bioinformatics.sdstate.edu/idep/), and Gene Ontology (http://geneontology.org).

## Source data

Source Data
