# Supplementary material for: Myeloid Krüppel-like factor 2 is a critical regulator of metabolic inflammation
Source: Nat Commun. 2020 Nov 18;11:5872. doi: 10.1038/s41467-020-19760-3 (PMC7674440; doi:10.1038/s41467-020-19760-3)
Supplement: Supplementary file 1 — Supplementary Information [file 41467_2020_19760_MOESM1_ESM.pdf]

SUPPLEMENTARY FIGURES FOR:

"Myeloid Krüppel-like factor 2 is a critical regulator of metabolic inflammation"

David R. Sweet[1,2,*], Neelakantan T. Vasudevan[1,*], Liyan Fan[1,2], Chloe E Booth[1] , Komal S Keerthy[1], Xudong Liao[1], Vinesh Vinayachandran[1], Yoichi Takami[3], Derin Tugal[4], Nikunj Sharma[5], E. Ricky Chan[6], Lilei Zhang[7], Yulan Qing[12,13], Stanton L Gerson[12,13], Chen Fu[8], Anthony Wynshaw-Boris[8], Panjamaporn Sangwung[1,9], Lalitha Nayak[10], Paul Holvoet[11,] Keiichiro Matoba[1], Yuan Lu[1,14], Guangjin Zhou[1], and Mukesh K. Jain[1,§]

INCLUDES:

8 Supplementary Figures and Legends

Supplementary Figure 1

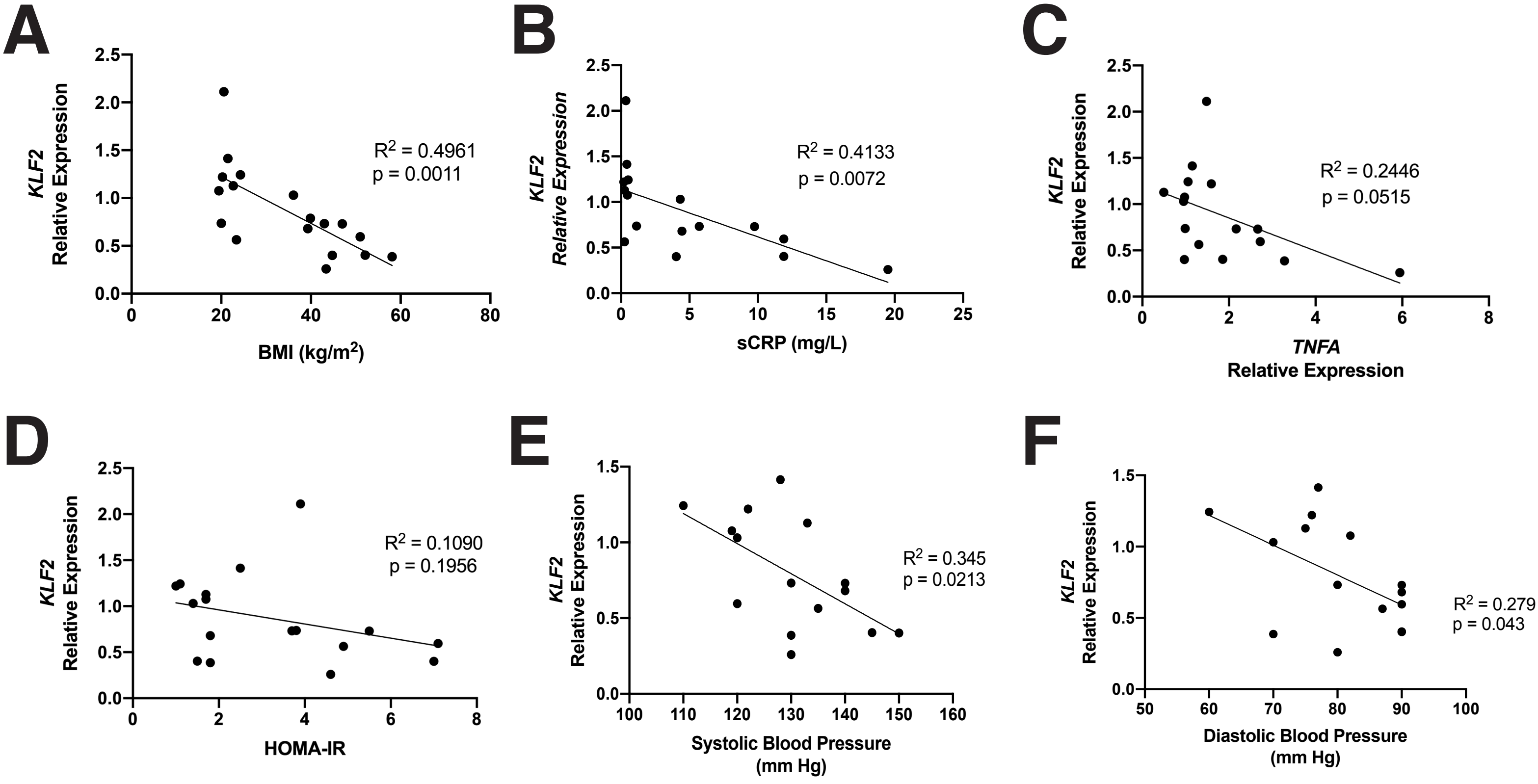

Supplementary Figure 1: KLF2 expression in patients correlated with (A) Body mass index (BMI), (B) soluble C-reactive protein (sCRP), (C) tumor necrosis factor alpha (TNFA) expression, (D) HOMA-IR insulin resistance score, (E) systolic blood pressure, and (F) diastolic blood pressure. n=15 human subjects. p-values listed are based on linear regression and whether slope is significantly non-zero.
Source data are provided as a Source Data file.

Supplementary Figure 2

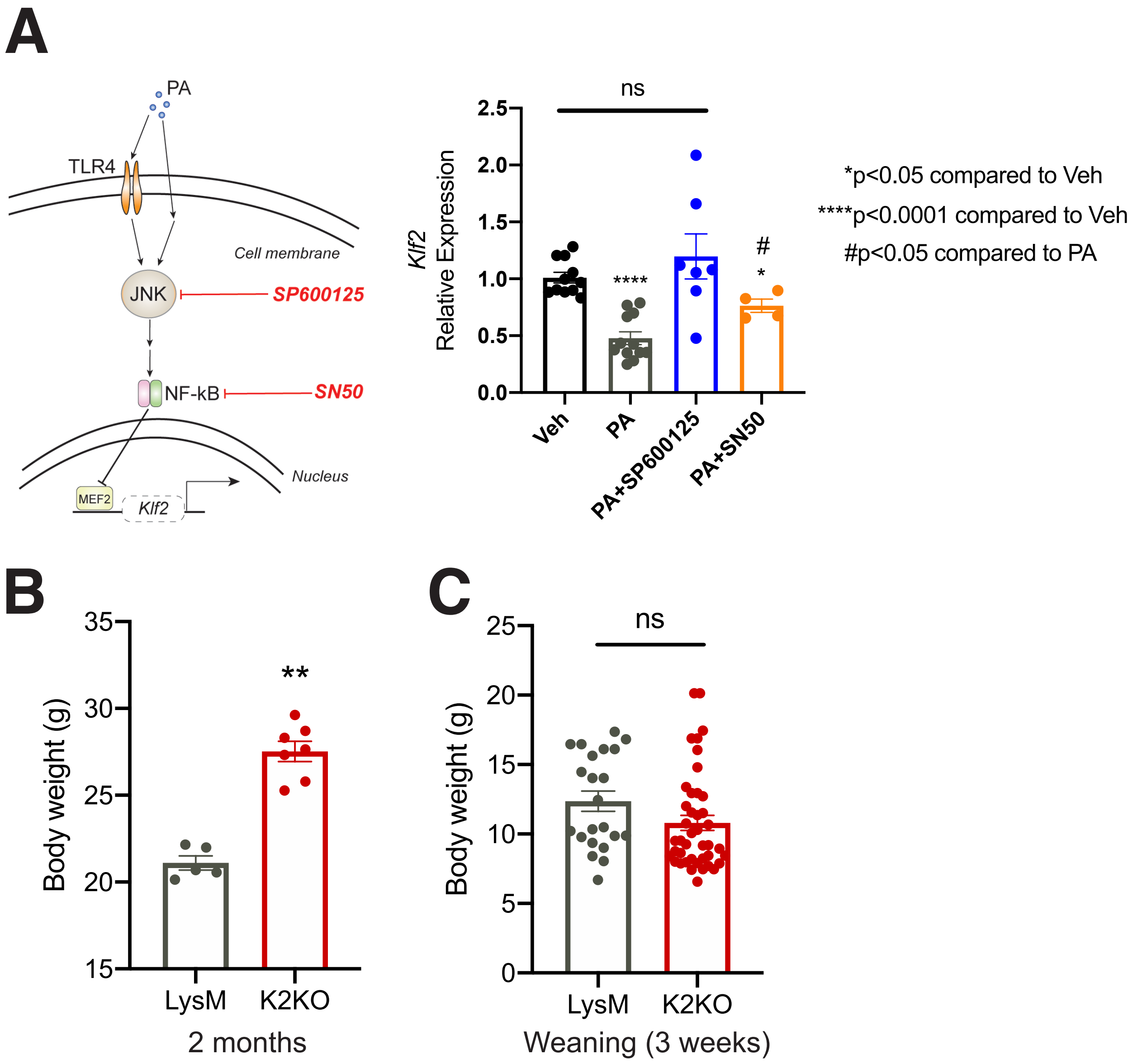

Supplementary Figure 2: (A) Schematic depicting pharmacologic targeting of upstream signaling of KLF2 expression. RAW264.7 cells treated with vehicle (Veh) or palmitic acid (PA) plus the addition of a JNK inhibitor (SP600125) or NF-kB inhibitor (SN50). $p < 0.0001$ PA vs Veh, $p = 0.0167$ PA+SN50 vs PA, n=4 independent experiments for PA+SN50. n=11 independent experiments for Veh and PA. n=7 independent experiments for PA+SP600125 (B) K2KO mice have significantly elevated body weight at 2 months of age (no dietary intervention), $p = 0.0025$, LysM n=5, K2KO n=7 biologically independent mice. (C) K2KO mice show no differences in body weight upon weaning at 3 weeks, LysM n=22, K2KO n=42 biologically independent mice. **$p<0.01$ by unpaired, two-tailed Student’s t test. Error bars represent SEM. Source data are provided as a Source Data file.

Supplementary Figure 3

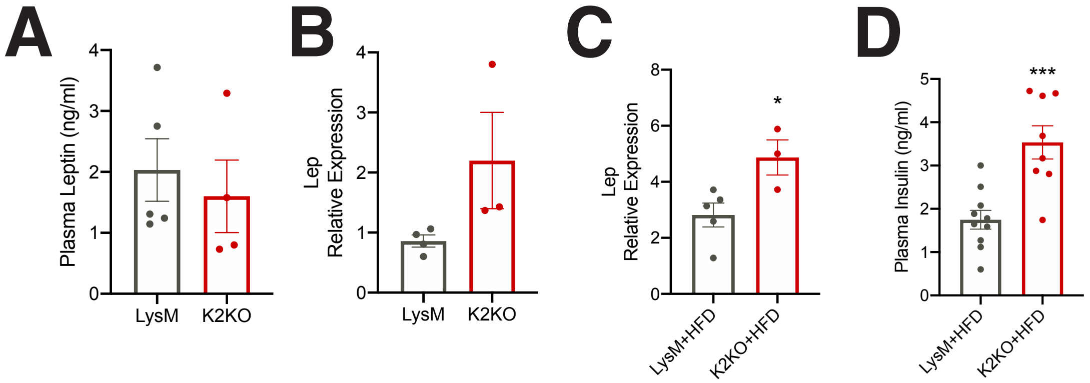

Supplementary Figure 3: (A) Comparison of plasma leptin levels between LysM and K2KO mice on one month CD illustrating no differences, LysM n=5, K2KO n=4 biologically independent mice. (B) Lep qPCR from WAT, one month CD, LysM n=4, K2KO n=3 biologically independent mice. (C) Lep qPCR from white adipose tissue (WAT), one month HFD, p = 0.0305 LysM n=5, K2KO n=3 biologically independent mice. (D) Plasma insulin levels after one month HFD, p = 0.0006, LysM n=10, K2KO n= 8 biologically independent mice. *p<0.05, **p<0.01, ***p<0.001 by unpaired, two-tailed Student’s t test. Error bars represent SEM. Source data are provided as a Source Data file.

Supplementary Figure 4

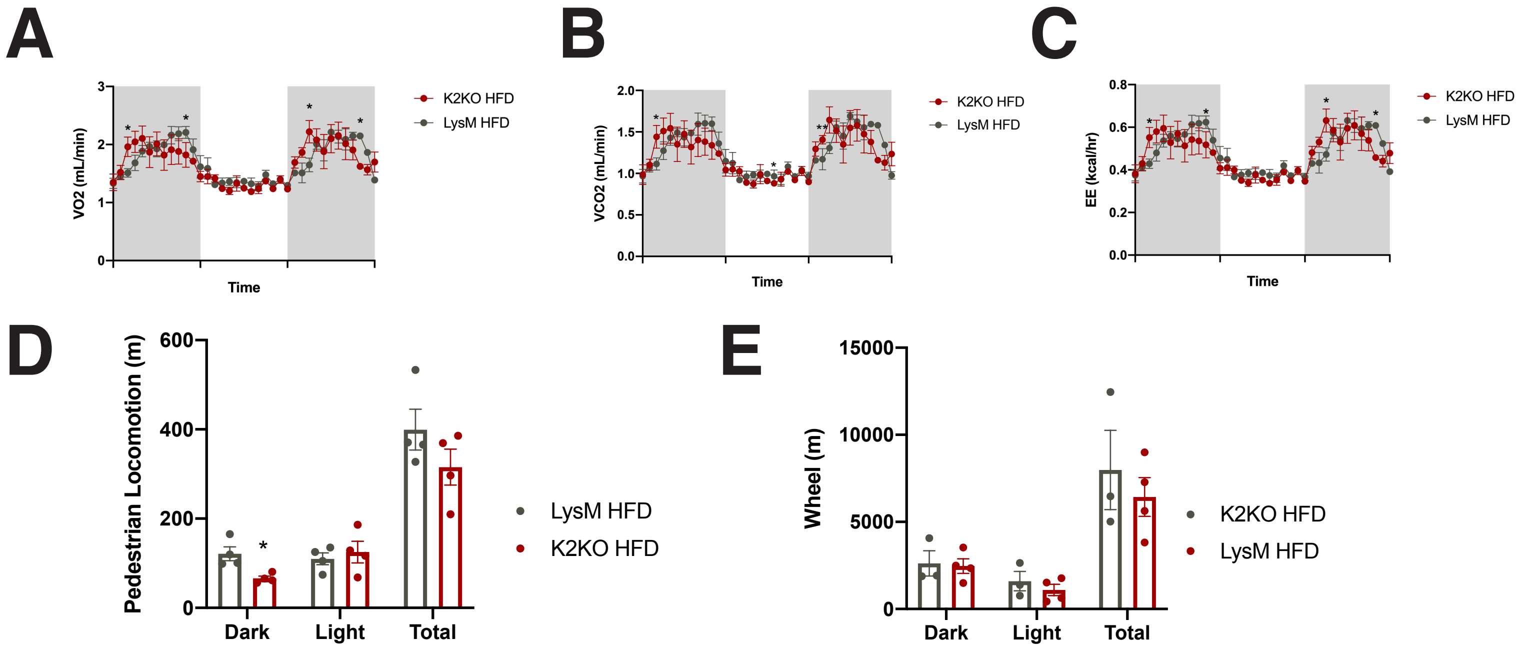

Supplementary Figure 4: Metabolic cage data of one month HFD-fed LysM and K2KO mice, n=4 biologically independent mice. (A) VO2 (p=0.022 20h, p=0.0491 4h, 0.0038 21h 0.0079 4h), (B) VCO2 (p=0.0237 20h, 0.019 21h, 0.0036 4h) (C) Energy Expenditure (EE) (p=0.0116 19h, 0.0168 20h, 0.0394 4h, 0.0019 20h, 0.0034 4h) (D) Pedestrian locomotion within cage, p= 0.0318 (E) Distance ran on wheel. *p<0.05 by unpaired, two-tailed Student's t test. Error bars represent SEM. Source data are provided as a Source Data file.

Supplementary Figure 5

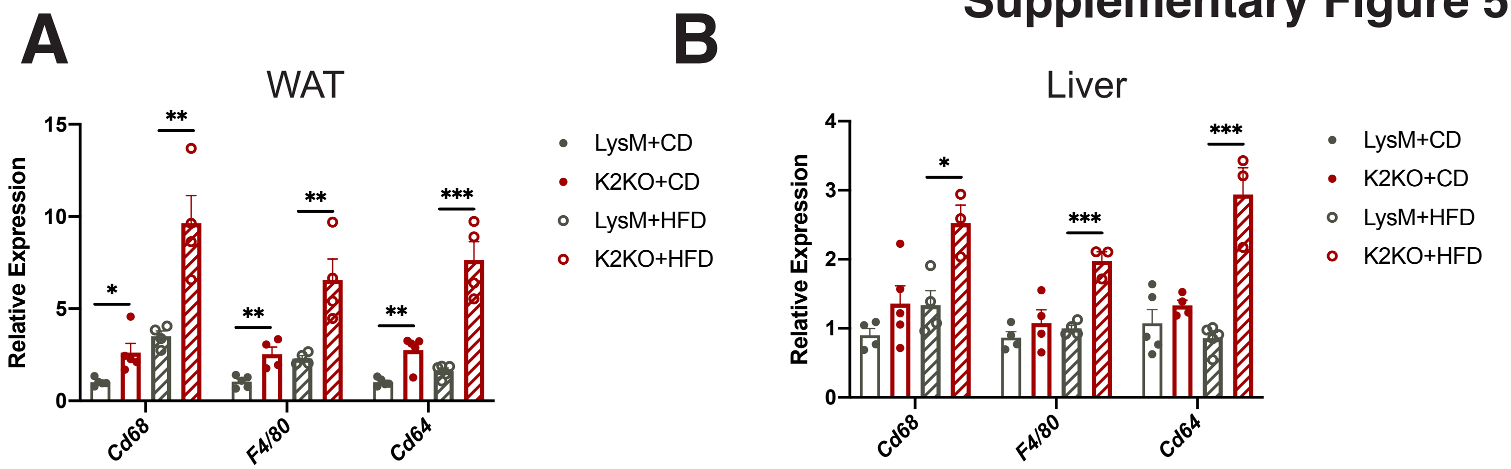

Supplementary Figure 5: qPCR of macrophage markers within (A) WAT and (B) liver. n=4 biologically independent mice. p values: WAT *Cd68*: CD = 0.0141, HFD = 0.0070, WAT *F4/80*: CD = 0.0061, HFD = 0.01, WAT *Cd64*: CD = 0.0021, HFD = 0.0003, Liver *Cd68*: HFD = 0.0162, Liver *F4/80*: HFD = 0.0005, Liver *Cd64*: HFD = 0.0005 by unpaired, two-tailed Student's t test, comparisons marked in figure.
Error bars represent SEM. Source data are provided as a Source Data file.

Supplementary Figure 6

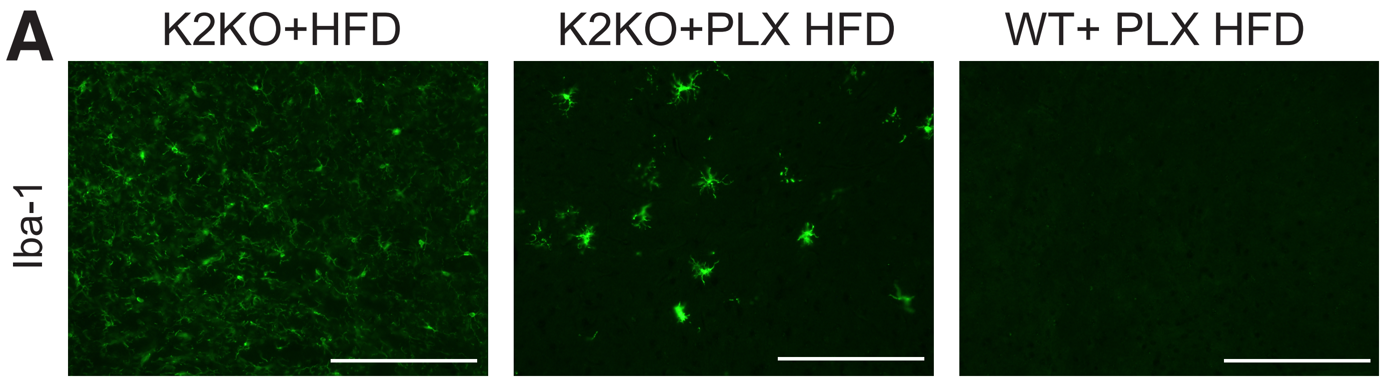

Supplementary Figure 6: (A) Representative images from Iba-1 stained hypothalami of one month HFD-fed K2KO mice, one month PLX HFD-fed K2KO mice (after three weeks CD PLX), and one month PLX HFD-fed WT mice as a control, scale bar = 200um

Supplementary Figure 7

A

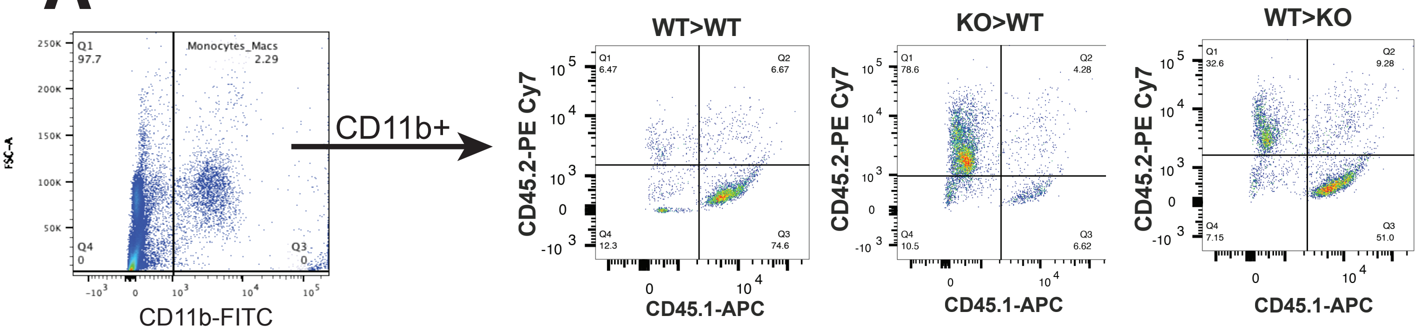

Supplementary Figure 7: (A) Representative flow cytometry scatter plots from the blood of chimeras demonstrating bone marrow engraftment after 2 months. Cells from each mouse were initially gated on CD11b to identify myeloid cells. From this population, CD45.2 vs CD45.1 populations were identified. K2KO mice express CD45.2, transplanted WT marrow into K2KO mice express CD45.1. WT marrow (CD45.1) was also transplanted into WT mice (CD45.2) as a control.

Supplementary Figure 8

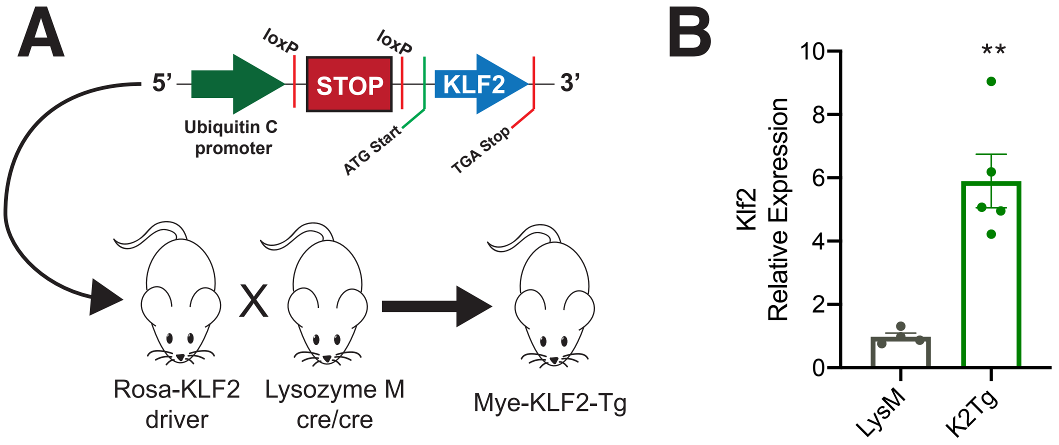

Supplementary Figure 8: (A) Schematic representation of myeloid KLF2 transgenic (K2Tg) mouse generation. (B) Confirmation of Klf2 overexpression in K2Tg bone marrow-derived macrophages, LysM n=4, K2Tg n=5 biologically independent mice, p = 0.0014 by unpaired, two-tailed Student's t test. Error bar represents SEM. Source data are provided as a Source Data file.
