# Supplementary material for: Myeloid Krüppel-like factor 2 is a critical regulator of metabolic inflammation
Source: Nat Commun. 2020 Nov 18;11:5872. doi: 10.1038/s41467-020-19760-3 (PMC7674440; doi:10.1038/s41467-020-19760-3)
Supplement: Supplementary file 2 — Reporting Summary [file 41467_2020_19760_MOESM2_ESM.pdf]

nature research

Corresponding author(s): Mukesh K. Jain, MD

Last updated by author(s): Aug 27, 2020

Reporting Summary

Nature Research wishes to improve the reproducibility of the work that we publish. This form provides structure for consistency and transparency in reporting. For further information on Nature Research policies, see our Editorial Policies and the Editorial Policy Checklist.

Statistics

For all statistical analyses, confirm that the following items are present in the figure legend, table legend, main text, or Methods section.

n/a	Confirmed	
☐	☒	The exact sample size (n) for each experimental group/condition, given as a discrete number and unit of measurement
☐	☒	A statement on whether measurements were taken from distinct samples or whether the same sample was measured repeatedly
☐	☒	The statistical test(s) used AND whether they are one- or two-sided<br>*Only common tests should be described solely by name; describe more complex techniques in the Methods section.*
☒	☐	A description of all covariates tested
☐	☒	A description of any assumptions or corrections, such as tests of normality and adjustment for multiple comparisons
☐	☒	A full description of the statistical parameters including central tendency (e.g. means) or other basic estimates (e.g. regression coefficient) AND variation (e.g. standard deviation) or associated estimates of uncertainty (e.g. confidence intervals)
☐	☒	For null hypothesis testing, the test statistic (e.g. F, t, r) with confidence intervals, effect sizes, degrees of freedom and P value noted<br>*Give P values as exact values whenever suitable.*
☒	☐	For Bayesian analysis, information on the choice of priors and Markov chain Monte Carlo settings
☒	☐	For hierarchical and complex designs, identification of the appropriate level for tests and full reporting of outcomes
☒	☐	Estimates of effect sizes (e.g. Cohen's d, Pearson's r), indicating how they were calculated

Our web collection on statistics for biologists contains articles on many of the points above.

Software and code

Policy information about availability of computer code

Data collection	ImageJ (v2.0.0-re-69/1.52p) for western blot quantification.
Data analysis	Statistics and graphing was performed using GraphPad Prism (v8) and Microsoft Excel (v16). Histology analysis utilized Fiji (Image J). Adobe Illustrator and Photoshop CC 2018 were used for image cropping and figure assembly. RNA seq was analyzed by Cufflinks v2.2.1 (including Cuffdiff).

For manuscripts utilizing custom algorithms or software that are central to the research but not yet described in published literature, software must be made available to editors and reviewers. We strongly encourage code deposition in a community repository (e.g. GitHub). See the Nature Research guidelines for submitting code & software for further information.

Data

Policy information about availability of data

All manuscripts must include a data availability statement. This statement should provide the following information, where applicable:

- Accession codes, unique identifiers, or web links for publicly available datasets
- A list of figures that have associated raw data
- A description of any restrictions on data availability

The RNA sequencing data discussed in this publication have been deposited in NCBI's Gene Expression Omnibus and are accessible through GEO Series accession number GSE149119. Source data for all applicable figures are provided in this paper. Databases and publicly available analysis software used in this manuscript include the Molecular Signature Database (MSigDB, https://www.gsea-msigdb.org/gsea/index.jsp), the Kyoto encyclopedia of genes and genomes (KEGG, https://www.genome.jp/kegg), iDEP.91 (http://bioinformatics.sdstate.edu/idep/), and Gene Ontology (http://geneontology.org).

Field-specific reporting

Please select the one below that is the best fit for your research. If you are not sure, read the appropriate sections before making your selection.

[x] Life sciences [ ] Behavioural & social sciences [ ] Ecological, evolutionary & environmental sciences

For a reference copy of the document with all sections, see nature.com/documents/nr-reporting-summary-flat.pdf

Life sciences study design

All studies must disclose on these points even when the disclosure is negative.

Sample size	No statistical methods were used to predetermine sample size (n). Number of sample was determined based on experimental approach, availability, feasibility required to obtain definitive results. Specifically, we utilized sample sizes previously described in the literature of similar experiments .
Data exclusions	No data excluded from analysis
Replication	All of experiments have been successfully repeated at least three times and/or with sufficient cells/animals per group to demonstrate statistical significance. All experiments were statistically analyzed.
Randomization	The samples or age-matched male mice were allocated into experimental groups randomly.
Blinding	The investigators were blinded to group allocation during data collection and/or analysis.

Reporting for specific materials, systems and methods

We require information from authors about some types of materials, experimental systems and methods used in many studies. Here, indicate whether each material, system or method listed is relevant to your study. If you are not sure if a list item applies to your research, read the appropriate section before selecting a response.

Materials & experimental systems

n/a	Involved in the study	
[ ]	[x]	Antibodies
[ ]	[x]	Eukaryotic cell lines
[x]	[ ]	Palaeontology and archaeology
[ ]	[x]	Animals and other organisms
[ ]	[x]	Human research participants
[x]	[ ]	Clinical data
[x]	[ ]	Dual use research of concern

Methods

n/a	Involved in the study	
[x]	[ ]	ChIP-seq
[ ]	[x]	Flow cytometry
[x]	[ ]	MRI-based neuroimaging

Antibodies

Antibodies used	rat monoclonal anti-CD107b (Mac-3) (550292,BD Biosciences, San Jose, CA, 550292) - Validated by BD Biosciences<br>rabbit polyclonal anti-Iba-1 (Wako, Richmond, VA, 019-19741) - Validated by Wako<br>goat polyclonal anti-IL-1b (R&D Systems, Minneapolis, MN AF-401) - Validated by R&D Systems<br>mouse monoclonal anti-Actin (Santa Cruz, Dallas, TX, sc-47778) - Validated by Santa Cruz<br>rabbit polyclonal anti-p-JNK (EMD Millipore, 07-175) - Validated by Millipore<br>rabbit polyclonal anti-SAPK/JNK (Cell Signaling, 9252S) - validated by CST
Validation	All antibodies were validated by vendors or other researchers. We based specificity on their provided data sheets. CD107b- Validated by BD Biosciences by staining paraffin-embedded normal mouse lung tissue. An isotype control was used as a negative control. Iba-1: Validated by staining frozen mouse brains. IL1b: Validated for western blot by R&D on mouse RAW264.7 cells +/- LPS treatment. Actin: Validated for western blot by Santa Cruz on NIH/3T3 cell lysate. pJNK: Validated by EMD Millipore for western blot using RAW246.7 cell lysate +/- phosphatase. SAPK/JNK: Validated by CST and community reviews for western blot.

Eukaryotic cell lines

Policy information about cell lines

Cell line source(s)	RAW 264.7 cell line was purchased from ATCC
Authentication	ATCC monitors morphology, recovery, and growth during the initial culturing of cell lines. Cytochrome C subunit I PCR was performed to confirm species.

Mycoplasma contamination	The cells were not tested for mycoplasma contamination.
Commonly misidentified lines (See ICLAC register)	No commonly misidentified cell lines were used in this study.

Animals and other organisms

Policy information about studies involving animals; ARRIVE guidelines recommended for reporting animal research

Laboratory animals	All mouse strains used in this study were male C57BL/6J mice. The strains include LysM Cre/Cre mice ('LysM'), LysM Cre/Cre KLF2 Flox/Flox (myeloid KLF2 KO mice, K2KO), LysM Cre/Cre Rosa26 Flox-Stop-Flox KLF2 (myeloid KLF2 transgenic mice, K2Tg), and wildtype C57BL/6J. Unless otherwise noted in the manuscript, all mice were started on experimental conditions at 2 months of age.
Wild animals	No wild animals were used in this study.
Field-collected samples	No field-collected samples were used in this study.
Ethics oversight	Case Western Reserve University IACUC approved and oversaw the study protocol

Note that full information on the approval of the study protocol must also be provided in the manuscript.

Human research participants

Policy information about studies involving human research participants

Population characteristics	The patient samples were collected from a sub-population of individuals in Hulsmans et al. 2012. In that study, the patient cohort comprised of 14 lean control (29% male) and 21 obese (33% male) individuals. We were blinded to the identities, sex, and age of the individuals whose samples we acquired. All participants were without symptoms of clinical atherosclerotic cardiovascular disease per the authors in the original study.
Recruitment	From the original study (Hulsmans et al, 2012): All human participants gave written informed consent. Samples were collected throughout one year at the Division of Endocrinology at KU Leuven.
Ethics oversight	This study complies with the Declaration of Helsinki and was originally overseen by the Medical Ethics Committee of KU Leuven

Note that full information on the approval of the study protocol must also be provided in the manuscript.

Flow Cytometry

Plots

Confirm that:

- [x] The axis labels state the marker and fluorochrome used (e.g. CD4-FITC).
- [x] The axis scales are clearly visible. Include numbers along axes only for bottom left plot of group (a 'group' is an analysis of identical markers).
- [x] All plots are contour plots with outliers or pseudocolor plots.
- [x] A numerical value for number of cells or percentage (with statistics) is provided.

Methodology

Sample preparation	Blood was drawn from mice via retroorbital bleed (50ul) and incubated with antibody cocktail at 4deg for 30min in FACS buffer.
Instrument	BD Biosciences LSR 2
Software	Flowjo (v9)
Cell population abundance	Myeloid cells were broadly defined as CD11b+. These cells accounted for about 2-6%. Because we were looking only at the presence of CD45 isoforms, we did not do additional gating to increase purity of specific populations.
Gating strategy	Cells from each mouse were initially gated on CD11b to identify myeloid cells. From this population, CD45.2 vs CD45.1 populations were identified. Cell populations were not compared between mice and flow cytometry was only used to confirm bone marrow engraftment for bone marrow transplant experiment.

- [x] Tick this box to confirm that a figure exemplifying the gating strategy is provided in the Supplementary Information.
